# Effects of Partial Replacement of Alfalfa Hay with Alfalfa Silage in Dairy Cows: Impacts on Production Performance and Rumen Microbiota

**DOI:** 10.3390/ani15182748

**Published:** 2025-09-19

**Authors:** Tian Xia, Zixin Liu, Ziyan Yang, Aoyu Jiang, Chuanshe Zhou, Zhiliang Tan

**Affiliations:** 1 State Key Laboratory of Forage Breeding-by-Design and Utilization, Institute of Subtropical Agriculture, Chinese Academy of Sciences, Changsha 410125, China; xiat9797@163.com (T.X.); lzx@isa.ac.cn (Z.L.); jiangaoyu21@mails.ucas.ac.cn (A.J.); zltan@isa.ac.cn (Z.T.); 2 University of Chinese Academy of Sciences, Beijing 101408, China; 3 Hunan Provincial Key Laboratory of Animal Nutritional Physiology and Metabolic Process, National Engineering Laboratory for Pollution Control and Waste Utilization in Livestock and Poultry Production, Institute of Subtropical Agriculture, Chinese Academy of Sciences, Changsha 410125, China; 15544552854@163.com; 4 College of Animal Science and Technology, Ningxia University, Yinchuan 750021, China; 5 Yuelushan Laboratory, Changsha 410128, China

**Keywords:** dairy cow, alfalfa silage, alfalfa hay, production performance, rumen microbiota

## Abstract

As an important feed source for ruminants, alfalfa’s rational and efficient utilization is of great significance for the production and economic benefits of pastures. This study focuses on Sanhe dairy cows and includes a control group (CON group, alfalfa in the diet is hay) and an experimental group (AS group, alfalfa silage partially replaces alfalfa hay of equal dry weight). The feeding experiment lasted for 60 days. The results revealed that, compared with the CON group, the AS group exhibited increased milk yield, milk protein, and milk fat. There were no significant differences in apparent digestibility, serum biochemical indicators, and volatile fatty acid (VFA) levels between the two groups. However, the microbial composition of the rumen differed significantly between the two groups of cows based on β-diversity. On the genus level, compared with the CON group, the relative abundance of *Erysipelatoclostridium*, *Pseudoflavonifractor*, and *Candidatus Saccharimonas* in the AS group was significantly reduced. In summary, partially replacing alfalfa hay with alfalfa silage feed is beneficial for improving the production performance of cows and changing rumen microbial diversity. These findings provide a basis for the effective utilization of alfalfa.

## Simple Summary

Alfalfa (*Medicago sativa* L.) is one of the major high-quality forages for ruminants and is an ideal feed source for high-yielding dairy cows. The currently common forms of utilization are alfalfa hay and alfalfa silage. However, there is currently limited research on the effects of alfalfa silage partially replacing alfalfa hay in dairy cows. Our research has found that partially replacing alfalfa hay with alfalfa silage feed is beneficial for improving the production performance of dairy cows and altering rumen microbial diversity. This provides a reliable source of data for future practical production.

## 1. Introduction

Alfalfa (*Medicago sativa* L.) is one of the major high-quality forages for ruminants because of its high protein content, and it has desirable characteristics as a feed source for high-producing dairy cows [1]. Furthermore, alfalfa is a vital source of energy for ruminants due to its high nutrient content, which includes vitamins, minerals, and crude fiber. Alfalfa has attracted considerable attention and widespread cultivation because of its growing significance in animal husbandry [2]. The development of human health cannot be separated from high-quality dairy products. Correspondingly, the scale of dairy farming and the number of dairy cows are constantly increasing. Consequently, there is a growing need for premium alfalfa in the husbandry of ruminant animals. At the moment, alfalfa hay and silage are the most widely used types. There are some variations in the nutritional value and feeding effects of alfalfa hay and silage on cows because of the diverse production methods.

Alfalfa silage feed has high digestible energy, and nutrients such as vitamin B and vitamin C are well preserved [3]. High-quality alfalfa silage can have a crude protein level of over 18%, as well as enhanced rumen-degradable protein and organic acid contents, both of which can increase the amount of nutrients available to ruminants [4]. Research has shown that, compared with feeding alfalfa hay, alfalfa silage results in a higher degradation rate of crude protein in the rumen [5]. In addition, alfalfa silage improves the taste of the feed during fermentation, and compared with alfalfa hay, alfalfa silage has better palatability [6,7]. A study on sheep showed that feeding silage significantly increased feed intake and improved the digestibility of dry matter and crude protein [8]. Alfalfa silage can increase the financial benefits of pastures because of its comparatively easy production process, which enables greater leaf utilization and minimizes losses. Alfalfa silage, however, necessitates rigorous fermentation control. At the same time, it is impossible to overlook the severe storage and transportation limitations of silage feed.

Alfalfa hay is currently the main form of preparation for alfalfa [9]. Hay feed often has a higher content of crude fiber [10]. It has been shown in ruminants that the proportion of Firmicutes increased with the crude fiber content in hay [11]. In addition, research has shown that the dry matter intake (DMI) of cows fed alfalfa hay was greater than that of cows fed alfalfa silage [5]. Moreover, during the drying process of alfalfa hay, there is less loss of antioxidants [12]. However, the production process of hay may lead to the loss of some water-soluble vitamins [13]. Although alfalfa hay is more conducive to pasture storage and has a higher protein content when compared with alfalfa silage, alfalfa hay has disadvantages such as poor palatability, low digestion and utilization rate, and higher cost.

The rumen is the most critical organ among ruminants, serving as a primary site of feed conversion, with rumen microbiota playing a key role in this process. The digestion and absorption of hay and silage feed in the rumen mainly depend on the action of rumen microorganisms. Both feeds can be fermented to produce volatile fatty acids (VFAs), approximately 50~85% of which are directly transported and absorbed by rumen epithelial cells as the main energy source for ruminants [14,15]. Cellulose and hemicellulose are the main components of alfalfa hay and silage. Compared with hay, alfalfa silage contains more water-soluble carbohydrates, which are the main substrates for rapid rumen fermentation [16,17]. The VFAs, acetate, and β-hydroxybutyric acid produced by rumen microbial fermentation of carbohydrates are directly absorbed by the gastric mucosa into mammary tissue and serve as precursor substances for milk fat synthesis [18]. However, rapid fermentation of silage may increase the risk of rumen acidosis [16]. The decomposition rate of cellulose and hemicellulose in hay is slow, and the production rate of VFAs is consequently low—this low VFAs production rate helps avoid the rapid accumulation of acids in the rumen [19]. Since alfalfa silage and alfalfa hay are distinct rumen fermentation substrates, they can influence the fermentation processes of rumen microbes and, consequently, the productivity of dairy cows. However, dairy cow performance can be improved by strategically combining hay and silage in their diet to optimize rumen fermentation efficiency as well as nutrient absorption and utilization.

Alfalfa silage and alfalfa hay each possess unique advantages. Combining these two feeds has the potential to improve the balance of protein and energy in dairy cow diets [20]. Moreover, this combination exhibits potential benefits in enhancing feed palatability, increasing dairy cow feed intake, and optimizing rumen fermentation [21,22]. Luo et al.’s study demonstrated that when corn silage and alfalfa hay were combined, dairy cows exhibited a higher dry matter digestibility and fiber digestibility compared with those in the group using fed corn silage alone. This difference may be attributed to the larger rumen volume, which is promoted by the higher neutral detergent fiber (NDF) content of alfalfa hay [23]. Cui et al. demonstrated that when wheat silage was used to partially replace oat hay (at replacement ratios of 36% or 64%), relative to the group fed oat hay alone, the concentrations of volatile fatty acids and microbial crude protein in lambs were significantly elevated, and the abundance of cellulolytic bacteria was also enhanced. This provides additional energy for ruminants. This phenomenon may be attributed to the increased fiber digestibility and a broader spectrum of nitrogen sources [24]. Furthermore, appropriate silage-to-hay ratios and effective alfalfa processing technologies enhance the economic benefits of grasslands and promote the sustainable development of animal husbandry. However, current research on the effects of alfalfa silage partially replacing alfalfa hay on dairy cows remains limited, and studies exploring the differences in nutritional or functional properties between these two forages are also scarce. Therefore, this study aims to investigate the effects of alfalfa silage partially replacing alfalfa hay on the production performance, serum biochemical indices, and rumen microbiota of dairy cows, thereby providing reliable data support for future practical animal production.

## 2. Materials and Methods

### 2.1. Animals, Experimental Design, and Diets

All procedures for animal experimentation were carried out according to guidelines approved by the Animal Care Committee, Institute of Subtropical Agriculture, Chinese Academy of Sciences (ISA-2022-0083).

Hulunbuir Agricultural Reclamation Xiertala Farm and Ranch Co. Ltd. (Hulunbuir City, China) provided the Sanhe dairy cows used in this study. A total of 20 Sanhe dairy cows with a parity of 2 and in mid-lactation were randomly divided into two groups, with 10 cows per group. Based on previous research data, milk yield was used as the primary index; the sample size required to achieve 80% statistical power was 8 cows per group, calculated using G*Power software (3.1.9.7). Therefore, considering potential model construction failure and animal mortality, 10 cows were selected for this experiment. The experimental group (AS group) received 3.0 kg of alfalfa silage to replace 1.5 kg of alfalfa hay on an equal dry matter basis, while the control group (CON group) was fed a basal diet containing 2.5 kg of alfalfa hay. The composition and nutritional levels of the diets are presented in Table 1. A 15-day pre-feeding period and a 45-day formal feeding period were included in the 60-day experimental period. In accordance with standard production protocols, each cow was housed individually in a pen. The cowshed was equipped with an outdoor exercise area and indoor fans. The experiment was conducted from July to September. The sprinkler system was used to cool the cows when the ambient temperature became excessively high. Cows were fed three times daily (6:30, 14:00, and 20:30). The staff cleaned up the remaining feed and recorded the daily feed intake before feeding them the next morning. The cows were allowed ad libitum access to drinking water.

### 2.2. Sample Collection

Total mixed ration (TMR) samples were collected daily throughout the experimental period. Following the completion of the experiment, the TMR samples were proportionately mixed to determine the nutritional composition.

Milk samples were collected from the dairy cows in this experiment following the completion of the experiment. In total, 40 aliquot mL of milk samples were taken from each cow in the morning, lunchtime, and evening in a 4:3:3 ratio by volume, and they were placed into a centrifuge tube containing potassium dichromate preservative. For the determination of milk composition, the samples were kept in a refrigerator at 4 °C.

The rectal sampling method was used to collect 300–500 g of fecal samples once in the morning and once in the evening, which was performed daily from the 43rd to 45th day of the formal experimental period. After evenly mixing 200 g of each cow’s fecal sample, 20 mL of 10% H_2_SO_4_ was added for nitrogen fixation. The treated fecal samples were then stored in a refrigerator at −20 °C until subsequent testing.

Before the morning feed on the final day of the formal experimental period, 10 mL of blood was collected from the tail vein of each cow. It was then allowed to stand for 2 h, and then centrifuged at 3000 rpm for 15 min at 4 °C. The resulting supernatant was aspirated into two mL centrifuge tubes, which were then kept at −20 °C until subsequent testing.

Rumen fluid was collected from the dairy cows before the morning feed on the 45th day of the formal experimental period. A rumen fluid sampler with a gastric tube was first inserted into the dairy cow’s mouth; subsequently, 100 mL of rumen fluid was aspirated. The first 50 mL of the aspirated rumen fluid was discarded to avoid saliva contamination. The remaining collected rumen fluid was filtered to remove solid residues, then aliquoted into 10 mL centrifuge tubes. These tubes were immediately transferred to a liquid nitrogen tank for rapid freezing and subsequent storage until testing.

All sample collection procedures were conducted under the premise of ensuring the health of the experimental cows. Feces from the cows were collected via rectal sampling. Blood was collected from the veins at the base of the cow’s tail. The rumen fluid sampler equipped with a gastric tube was employed to collect rumen fluid via the cow’s mouth. All sample collection activities complied with the relevant animal ethical standards. The specific operational procedures were consistent with those described in previous studies [25].

### 2.3. Analytical Procedures

#### 2.3.1. Diets, Feces, and Apparent Digestibility

The determination methods for feed and fecal components were consistent with those previously employed by Liu et al. [26]. The apparent digestibility was determined using the method previously employed by Van Keulen et al. [27], employing acid-insoluble ash in feces and feed as an endogenous indicator. The formula was as follows:

$$Apparent\;digestibility\;(\%)=[1-\frac{A_{d}\times N_{f}}{A_{f}\times N_{d}}]\times 100$$

In the formula:

*A_d_* and *A_f_* represent the acid-insoluble ash (AIA) content in the diet and feces, respectively (g/kg);

*N_d_* and *N_f_* represent the nutrient content of a certain nutrient in the diet and feces, respectively (g/kg).

#### 2.3.2. Serum Biochemical Parameters

Serum biochemical parameters were measured using an automatic biochemical analyzer (Hitachi 7600; Hitachi, Tokyo, Japan), including total protein (TP, A045-2-2), albumin (ALB, A028-1-1), globulin (GLB), alanine aminotransferase (ALT, C009-3-1), aspartate aminotransferase (AST, C010-3-1), alkaline phosphatase (ALP, A059-1-1), urea nitrogen (BUN, C013-1-1), glucose (GLU, F006-1-1), triglycerides (TG, A110-1-1), and total cholesterol (T-CHO, A111-1-1). In addition, serum immunoglobulin G (IgG, H106-1-1), immunoglobulin A (IgA, H108-1-2), immunoglobulin M (IgM, H109-1-2), interferon-γ (IFN-γ, H025-1-2), interleukin-1β (IL1β, H002-1-2), interleukin-2 (IL-2, H003-1-1), interleukin-6 (IL-6, H007-1-2), interleukin-8 (IL-8, H008-1-1), and tumor necrosis factor-alpha (TNF-α, H052-1-2) concentrations were all measured using commercial reagent kits (Nanjing JianCheng Bioengineering Institute, Nanjing, China). Refer to the commercial reagent kit manual for the determination method.

#### 2.3.3. Milk Composition

A Fossomatic FC Type 79910 equipment (Foss Electric, Hillerod, Denmark) was used to measure the somatic cell count, and a Basic Unit MilkoScan FT+200 Type 76150 device was used to assess the quality of the milk samples [28]. The 4% FCM (Fat-Corrected Milk) and ECM (Energy-Corrected Milk) yields were calculated [25,29]. The formulas were as follows:

4% FCM (Fat−Corrected Milk)=milk yield (kg/d)×[milk fat (%)×0.15+0.4]

ECM (Energy−Corrected Milk)                 =milk yield (kg/d)×[0.0929×milk fat (%)                     +0.0547×milk protein (%)+0.0395×milk lactose (%)]

#### 2.3.4. Measurement of Rumen Fermentation Parameters

Rumen fluid was collected and then centrifuged at 4 °C and 10,000× *g* for 10 min. An aliquot (1.5 mL) of the supernatant was taken and mixed with 0.15 mL of 25% metaphosphoric acid for fixation. This mixture was then used for the determination of volatile fatty acids (VFA) [30]. The concentrations of microbial crude protein (MCP), ammonia nitrogen (NH_3_-N), and VFA concentrations in the rumen fluid were determined using the methods described by Wang [31], Weatherburn [32], and Zinn [33], respectively.

#### 2.3.5. DNA Extraction and 16S rRNA Gene Sequencing

Following the manufacturer’s instructions, microbial DNA was isolated from rumen fluid samples using the E.Z.N.A.^®^ Soil DNA Kit (Omega Bio-tek, Norcross, GA, USA). The full-length segment of the 16S rRNA gene was subjected to a polymerase chain reaction (PCR) using primers 27F (5′-AGAGTTTGATCMTGGCTCAG-3′) and 1492R (5′-ACCTTGTTACGACTT-3′) [34,35]. The 25 μL PCR amplification system contained Q5 DNA Polymerase 0.25 μL, dNTP 2.5 mM 2 μL, Forwardprimer 10 μM 1 μL, Reverseprimer 10 μM 1 μL, DNA Template 2 μL, ddH_2_O 8.75 μL, 5 × reaction buffer 5 μL, and 5 × GC buffer 5 μL. Following their extraction from 2% agarose gels, amplicons were purified using the AxyPrep DNA Gel Extraction Kit (Axygen Biosciences, Union City, CA, USA) [36]. As directed by the manufacturer (Pacific Biosciences, Shanghai, China), SMRTbell libraries were created from the amplified DNA using blunt ligation. Shanghai Biozeron Biotechnology Co. Ltd. (Shanghai, China) carried out all amplicon sequencing. The SMRT Link Analysis software version 9.0 was used to process PacBio raw data and produce demultiplexed circular consensus sequence (CCS) reads.

With a 98.65% similarity criterion, OTUs were grouped using UPARSE 7.1 (http://drive5.com/uparse/) (accessed on 8 December 2022), and chimeric sequences were found and removed using UCHIME. The RDP Classifier (https://sourceforge.net/projects/rdp-classifier/) (accessed on 8 December 2022) was used to analyze the phylogenetic relationship of each 16S rRNA gene sequence at a 70% confidence level against the Silva (SSU132)16S rRNA database [37]. The beta diversity was determined using weighted and unweighted UniFrac distance matrices, and alpha and beta diversity analysis were performed using the table acquired from OTU, and then using R software (V3.1) to generate a principal coordinate analysis graph (PCoA) [37]. To compare the variations in relative bacterial abundance between the two groups, linear discriminant analysis effect size (LEfSe) was used. Significant differences between the groups are indicated by *p* < 0.05 and linear discriminant analysis (LDA) > 2 [38]. For more detailed steps, refer to previous research in our laboratory [39].

#### 2.3.6. Correlation Analysis

The linkET R package (0.0.3) [40] was then used to visualize these microbial species and examine their associations with milk yield, milk protein, milk fat, and milk lactose using the Mantel test method. We conducted Spearman correlation tests after linearly fitting the relative abundance of rumen microbes using the ggplot2 R software (version 4.1.2).

### 2.4. Statistical Analysis

The Shapiro–Wilk normality test of all data was conducted using SPSS 24.0 statistical software. All data conformed to a normal distribution. The analysis was performed using Student’s *t*-test using SPSS 24.0 statistical software. When *p* < 0.05, the data were statistically significant. There was no significant difference in the data when *p* > 0.05. When 0.05 < *p* < 0.1, a trend was observed in the data. All data were presented as means and standard error of the mean (SEM).

## 3. Results

### 3.1. Lactation Performance, Milk Quality, and Apparent Digestibility

The effect of the partial replacement of alfalfa hay with alfalfa silage on milk yield, milk quality, and apparent digestibility of nutrients in Sanhe dairy cows was presented in Table 2. There were no significant differences in milk yield, milk quality, and apparent digestibility between the CON and AS groups (*p* > 0.05). However, compared with the CON group, the AS group showed an increasing tendency in milk yield (*p* = 0.092). The 4% FCM and ECM in the AS group were significantly higher than those in the CON group (*p* < 0.05). Compared with the CON group, the AS group showed increases in milk yield, milk protein, and milk fat levels by 9.73%, 2.71%, and 5.70%, respectively.

### 3.2. Serum Biochemical Indicators

The effect of the partial replacement of alfalfa hay with alfalfa silage on serum biochemical indicators is presented in Table 3. Compared with the CON group, the serum TP and TG levels in the AS group increased by 2.70% and 8.07% (*p* > 0.05), respectively, while the serum ALP level in the AS group dropped by 7.06% (*p* > 0.05). There was no significant difference in serum biochemical indicators between the two groups (*p* > 0.05).

### 3.3. Serum Immune Parameters

The effect of the partial replacement of alfalfa hay with alfalfa silage on serum immune indicators of Sanhe dairy cows is presented in Table 4. Compared with the CON group, the content of IgG in the serum of cows in the AS group was significantly reduced (13.59%, *p* < 0.05), while the content of IL-6 in the AS group was significantly higher than that in the CON group (39.18%, *p* < 0.05). There was no significant difference in other immune-related indicators between the two groups (*p* > 0.05).

### 3.4. Rumen Fermentation Parameters

The effect of the partial replacement of alfalfa hay with alfalfa silage on rumen fermentation parameters of Sanhe dairy cows was presented in Table 5. Compared with the CON group, the MCP content in the AS group was significantly reduced (*p* < 0.05). Compared with the CON group, the AS group showed a 4.12% increase in acetic acid content and a 12.45% decrease in propionic acid content (*p* > 0.05), but there was no significant change. There was no significant change in other indicators as well (*p* > 0.05).

### 3.5. Rumen Microbial Richness and Diversity

**Figure 1:**
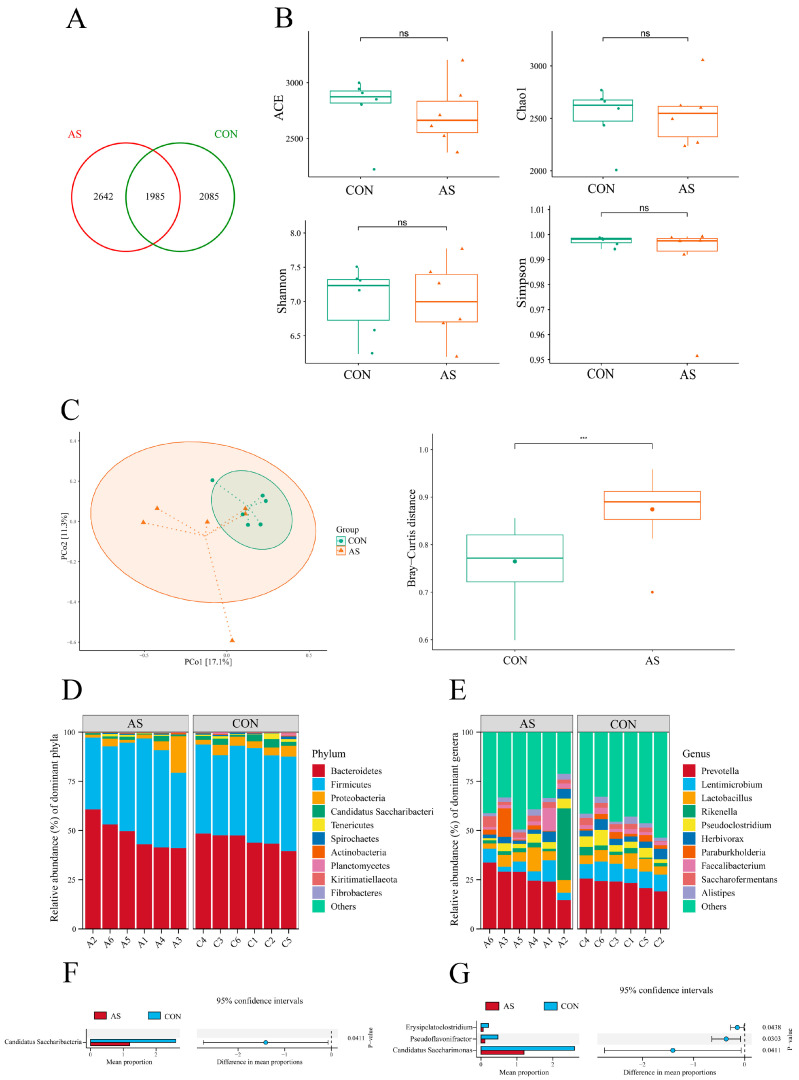
The effect of alfalfa silage partially replacing alfalfa hay on rumen microbiota of dairy cows. (**A**) Venn diagram of rumen microbiota on OTU level in the two groups. (**B**) Alpha diversity. (**C**) Beta diversity. (**D**) Changes and differences in microbiome on phylum level. (**E**) Changes and differences in microbiome on genus level. (**F**) The microbial communities with significant differences at the phylum level. (**G**) The microbial communities with significant differences at the genus level. “ns” indicates no significant difference between the two groups. “***” indicates that there is a highly significant difference between the two sets of data (*p* < 0.001).

A total of 1985 OTUs were present in the two groups, 2085 of which were specific to the CON group and 2642 to the AS group (Figure 1A). Compared with the CON group, there were no significant differences in the ACE index, Chao 1 index, Shannon index, and Simpson index in the AS group (*p* > 0.05, Figure 1B). The plot of principal coordinates analysis revealed clear clustering of the CON group and the AS group, with obvious separation between them. And there was a significant difference in the Bray–Curtis distance between the two groups (*p* < 0.05, Figure 1C). *Bacteroidetes* and *Firmicutes* accounted for more than 90% of the microbiota in each group, making them the dominant phyla in both groups (Figure 1D). There were notable variations across the groups: *Candidatus Saccharibacteria* relative abundances were larger in the CON group (*p* < 0.05, Figure 1F).

In terms of genus composition, both groups mainly had *Prevotella*, *Lentimicrobium*, and *Lactobacillus* as the dominant bacterial groups (Figure 1E). Compared with the CON group, the relative abundance of *Erysipelatoclostridium*, *Pseudoflavonifractor*, and *Candidatus Saccharimonas* in the AS group was significantly reduced (*p* < 0.05, Figure 1G).

### 3.6. Correlation Analysis

**Figure 2:**
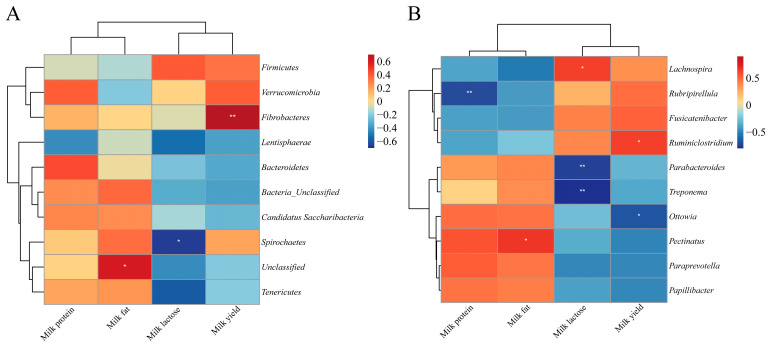
Correlation analysis between rumen microbiota and milk yield, milk protein, milk fat, and milk lactose. (**A**) Correlation analysis at microbial phylum level. (**B**) Correlation analysis at microbial genus level. The red block represents positive correlation, and the blue block represents negative correlation. “*” Indicates significant difference (*p* < 0.05), “**” indicates extremely significant difference (*p* < 0.01). The mean OTUs for *Fibrobacteres* phylum were 0.5 for samples in the CON group and 1.33 for samples in the AS group. The mean OTUs for *Ruminiclostridium* genus were 61.17 for samples in the CON group and 63.33 for samples in the AS group.

At the phylum level, there was a significant positive correlation between rumen *Fibrobacteres* and milk yield in cows (*p* < 0.01, Figure 2A). There was a significant negative correlation between *Spirochaetes* and lactose (*p* < 0.05, Figure 2A). At the genus level, significant positive correlations were observed between *Ruminiclostridium* and milk yield, *Pectinatus* and milk fat, as well as *Lachnospira* and lactose (*p* < 0.05, Figure 2B). However, there was a negative correlation between the relative abundance of *Ottowia* and milk yield (*p* < 0.05, Figure 2B). Meanwhile, there was a significant negative correlation between *Rubripirellula* and milk protein (*p* < 0.01, Figure 2B). In addition, *Parabacteroides* and *Treponema* were negatively correlated with lactose (*p* < 0.01, Figure 2B).

## 4. Discussion

### 4.1. Lactation Performance, Milk Quality, and Apparent Digestibility

Alfalfa hay is an important feed source in dairy farming. However, the cultivation of alfalfa is constrained by climatic and soil conditions (key environmental factors) [41], alongside rising import costs [42,43] and high processing costs [44]—factors that consequently pose significant challenges to dairy farming. Alfalfa silage and alfalfa hay each possess unique advantages. Therefore, this study aims to explore the potential impacts of the partial replacement of alfalfa hay with alfalfa silage on Sanhe dairy cows.

Alfalfa was one of the main high-quality roughages in the production of dairy cows, which can provide nutrition of high quality to improve milk quality and production [45]. According to the statistical analysis results, the milk yield of the cows in the CON and AS groups was 23.33 kg/d and 25.60 kg/d, respectively, at the end of the experimental period. Although there was no significant difference in milk yield between the two groups, the milk yield in the AS group showed an upward trend than that in the CON group, and 4% FCM production was significantly increased. The 4% FCM and ECM in the AS group were significantly higher than those in the CON group. This indicates an improvement in milk quality and an increase in feed utilization efficiency [46]. Silage retains more digestible nutrients during its fermentation process, particularly soluble sugars and starch. These substances were more easily degraded by ruminal microorganisms, thereby providing the dairy cow’s body with more energy and nutrients [47]. This may be the reason for the increase in 4% FCM and ECM in dairy cows. At the same time, the milk protein and fat content in the AS group also increased. The two groups’ apparent digestibility and milk quality, however, did not differ significantly. In contrast, previous studies have demonstrated that replacing chopped alfalfa hay with alfalfa silage tended to increase milk fat percentage and milk fat yield, with the milk protein content also increasing [4,48]. Compared with dairy cows given alfalfa hay, those fed alfalfa silage had a 15.45% higher milk fat content and a 12.31% higher milk fat output [20]. These were similar to the results of our study. According to earlier research, silage’s digestible energy value (per kilogram of dry matter) was almost 1.24 times higher than hay’s [49]. Therefore, one of the reasons for the relatively higher milk protein and milk fat contents in the AS group in this study may be that alfalfa silage had higher digestible energy—particularly ruminal digestible energy—compared with alfalfa hay, which in turn provided more energy for the synthesis of milk protein and milk fat in dairy cows. In addition, the fiber digestibility of silage is higher, which can better stimulate rumen fermentation and produce more precursors for milk fat synthesis [20]. Silage feed has good palatability [50] and can effectively preserve nutritional components [51,52], especially protein and energy, in the raw materials during the production process, which may partly explain the improved milk yield in cows.

### 4.2. Serum Biochemistry and Immune Parameters

Blood is an important medium for the transportation of nutrients in living organisms [53]. In ruminant animals, the blood system is closely involved in rumen fermentation, the absorption of rumen fermentation products, and milk synthesis. In this study, there was no significant difference in serum biochemical indicators between the two groups. A study of Holstein calves showed that changing the proportion of hay or silage in the diet did not significantly affect blood glucose, blood urea nitrogen, total protein, and globulin [54]. This was similar to our research. Silage had stable nutritional content, and its fermentation process can lower the feed’s pH value—thereby inhibiting the proliferation of pathogenic bacteria and reducing the production of potentially harmful substances [55]. This avoided additional metabolic burdens on the liver and blood system. This may partially explain why the partial replacement of alfalfa hay with alfalfa silage had no significant effect on serum biochemical parameters in the present study. In addition, determinations of immune-related indicators showed a significant decrease in IgG content and a significant increase in IL-6 content in the serum of the AS group. This observation suggested that the AS group may face potential risks of immunosuppressive or inflammation. Research has shown that improper storage of silage feed can lead to fungal spoilage and mycotoxin contamination [56]. Mycotoxins can activate the inflammatory response of the immune system, leading to the release of pro-inflammatory cytokines such as IL-6. Meanwhile, it can inhibit the differentiation of B lymphocytes and the production of antibodies, leading to a decrease in IgG synthesis [57].

### 4.3. Rumen Fermentation and Microorganisms

VFA, a crucial energy substrate created by microbial fermentation in the rumen, can supply 80% of ruminants’ energy needs [58]. The partial substitution of alfalfa silage for alfalfa hay exerted no significant effect on VFA. However, changes in the concentrations of acetic acid and propionic acid suggest that the rumen fermentation types of the CON and AS groups may be changing. Specifically, compared with the CON group, the AS group exhibited an increasing trend in acetic acid concentration and a decreasing trend in propionic acid concentration. Acetic acid is a precursor substance for milk fat synthesis [59]. This may be the reason why the milk fat levels in the AS group were relatively high. In addition, the MCP content in the AS group significantly decreased. Previous studies have shown that partially replacing alfalfa hay with corn silage decreases ruminal MCP production. This is because corn silage has a high organic acid content—particularly its lactic acid concentration—which may inhibit the activity and proliferation of rumen microorganisms, thereby subsequently reducing the digestibility of nutrients [60]. Therefore, the synthesis of MCP was reduced. Notably, the lower antioxidant content of the silage may also upset the redox balance in ruminal microbes, decreasing the percentage of nutrients directed towards microbial protein synthesis, since rumen bacteria were known to have a lower antioxidant capacity than aerobic bacteria [61].

Multi-omics research has shown that the rumen microbiota is essential for dairy cow performance. For ruminants, the rumen microbiota acts as a bioreactor that allows dairy cows to obtain nutrients from plant matter that was indigestible to humans [62]. In the present study, 16S rRNA gene sequencing revealed a significant difference in beta diversity analysis between the CON and the AS group. At the phylum taxonomic level, the *Bacteroidetes* and *Firmicutes* accounted for the largest proportion of the ruminal microbiota. This composition pattern was consistent with many relevant previous studies [63,64]. Numerous fiber-degrading bacteria found in the *Bacteroidetes* and *Firmicutes* may break down cellulose in the feed to create volatile fatty acids and are crucial to the rumen’s breakdown of carbohydrates, which supports ruminant growth and development [65]. There was no significant difference in the relative abundance of *Bacteroidetes* and *Firmicutes* between the CON and AS groups. This finding supports the normal digestion and utilization of silage or hay feed by cows. *Prevotella* was the primary prevalent bacteria in the CON and AS groups at the genus level. As a prominent genus in the ruminant digestive system, *Prevotella* is known to play crucial roles in the rumen ecology, including the synthesis of amino acids and short-chain fatty acids—such as propionate and acetate [66]. The abundance of *Prevotella* in the AS group (25.3%) was higher than that in the CON group (21.6%). Studies have shown that feeding black goats with cassava silage can increase the abundance of *Prevotella* in the rumen. This is similar to our research findings. During the ensiling process of alfalfa, its cell walls decompose, releasing more soluble sugars and thereby providing more fermentable substrate for *Prevotella*. This could explain the increased relative abundance of *Prevotella* in the AS group [67]. Partially replacing alfalfa hay with alfalfa silage increased the abundance of *Prevotella* and enhanced the degradation ability of the feed. In addition, the AS group significantly reduced the abundance of *Erysipelatoclostridium*, *Pseudoflavonifractor*, and *Candidatus Saccharimonas*. Studies have shown that *Erysipelatoclostridium* can cause severe infection in the host [68], while an increase in the abundance of *Pseudoflavonifractor* [69] and *Candidatus Saccharimonas* [70,71] was associated with host inflammation. The low content of fermentable carbohydrates (such as starch and water-soluble carbohydrates) in alfalfa hay leads to insufficient fermentation substrates for beneficial bacteria such as lactic acid bacteria in the rumen [72]. Reduced activity of beneficial ruminal bacteria may induce an imbalance in the rumen microbial community. This could explain the higher relative abundance of pathogenic bacteria in the CON group. Supplementing alfalfa silage in the AS group’s diet alleviated this phenomenon. Furthermore, these changes in specific microbial taxa may account for the significant difference in β-diversity between the two groups. In contrast, since the overall ruminal microbiota remained stable and the dominant bacterial phylum was similar between groups, this could explain the absence of a significant difference in α-diversity. These two phenomena are not contradictory. These results indicated that alfalfa silage alters the composition of rumen microbiota and is beneficial for the healthy development of dairy cows. According to Cui et al.’s study, the diet containing 100% wheat silage had a lower relative abundance of *Proteobacteria*, which are Gram-negative bacteria that include pathogens like *Salmonella* and *Escherichia coli*, compared with the group that used oaten hay as their only source of fodder [24]. Similarly, Yin et al. found that the inclusion of whole-plant corn silage in the feed can reduce the abundance of some species of pathogenic bacteria in pigs, thereby reducing the incidence of disease in the animals [73]. These findings from previous studies are consistent with the results of the present study. One plausible explanation for this consistency is that the anaerobic fermentation process of alfalfa silage produces large quantities of lactic acid and other organic acids, which exert an inhibitory effect on bacterial growth. In contrast, compared with alfalfa silage, alfalfa hay is more susceptible to contamination by microorganisms (e.g., molds) during storage, thereby reducing its nutritional value and increasing the risk of diseases in Sanhe dairy cows.

### 4.4. Correlation Analysis

The correlation analysis results demonstrate a statistically significant positive correlation between the phylum *Fibrobacteres* and the milk yield of Sanhe dairy cows. *Fibrobacteres* has been shown in previous studies to be the principal cellulolytic active bacteria in the rumen [74,75]. The fiber is broken down in the rumen and can be used to generate VFA, providing an energy source for ruminants [76]. This may explain the statistically significant positive correlation between *Fibrobacteres* and milk yield. In addition, previous research has found that a decrease in the relative abundance of the phylum *Fibrobacteres* is observed in ewes at parturition, indicating that *Fibrobacteres* may be highly sensitive to changes in rumen ecological conditions [77]. In this study, the partial substitution of alfalfa silage for alfalfa hay changed the microbial fermentation substrate and similarly altered rumen ecological conditions. This further explains the correlation between the changes in *Fibrobacteres* and milk yield in the AS group. Specifically, the average number of OTUs for *Fibrobacteres* in the AS group was higher than that in the CON group, representing a relative increase of 166%. Further studies are needed to clarify the specific mechanisms underlying the link between *Fibrobacteres* (e.g., its OTU diversity) and milk yield. At the genus level, a statistically significant positive correlation was observed between the relative abundance of *Ruminiclostridium* and milk yield. It has been shown that the function of *Ruminiclostridium* is related to the degradation of flavonoids and xyloglucans, among others [78]. Coumarin and vanillic acid, produced by the decomposition of flavonoids, have antioxidant and anti-inflammatory effects [79]. Xyloglucan decomposition produces xylose, and glucose can provide energy for the animal body [80]. They are all effective in improving ruminant health. The average number of OTUs for *Ruminiclostridium* was 3.53% higher in the AS group compared with the CON group. This may explain the reason for the significant positive correlation between *Ruminiclostridium* and milk yield in the AS group.

The primary limitation of this study is its small sample size. Therefore, this study should be considered a preliminary exploratory investigation. The core value of its findings lies in the proposal of hypotheses rather than their verification; these hypotheses can provide research directions and candidate targets for subsequent confirmatory studies conducted in larger-scale cohorts.

## 5. Conclusions

The partial replacement of alfalfa hay with alfalfa silage tends to increase the milk yield of Sanhe dairy cows and alter rumen microbial diversity. This observation may be attributed to enhanced microbial capacity for fiber degradation and utilization, thereby increasing digestible energy. However, the replacement of part of alfalfa hay with alfalfa silage may pose risks of immunosuppression or inflammation to dairy cows, and this replacement strategy also leads to a reduction in microbial crude protein. These potential issues warrant further investigation.

## Figures and Tables

**Table 1:** Composition and nutrient levels of the basal diet (DM basis) %.

Ingredients (%)	CON	AS
Cornsilage	35.74	35.74
Concentrated feed ^1^	39.73	39.73
Alfalfa hay	9.46	3.78
Alfalfa silage	0.00	5.68
Flakedcorn	8.70	8.70
Beetrootmeal	5.74	5.74
Bakingsoda	0.63	0.63
Total	100.00	100.00
Nutrient levels ^2^ (%)		
CP	14.89	14.83
EE	3.05	3.08
NDF	32.37	32.59
ADF	17.04	17.16
Ash	2.97	3.02

^1^ The concentrated feed for dairy cows during lactation was formulated by Xiertala first farm, with the following main ingredients: soya bean meal, DDGS, calcium phosphate, and premix. Premix composition per kilogram: 4500 mg of Fe, 1600 mg of Cu, 3000 mg of Mn, 5500 mg of Zn, 30 mg of Se, 20 mg of Co, 30 mg of I, 600 KIU of vitamin A, 200 KIU of vitamin D, 200 IU of vitamin E. ^2^ CP = crude protein; EE = ether extract; NDF = neutral detergent fiber; ADF = acid detergent fiber.

**Table 2:** The effect of the partial replacement of alfalfa hay with alfalfa silage on lactation performance, milk quality, and apparent digestibility of dairy cows.

Items	CON	AS	SEM	*p*-Value
Milk yield (kg/d)	23.33	25.60	0.897	0.092
Milk composition				
Milk protein (%)	3.39	3.49	0.040	0.146
Milk fat (%)	3.51	3.71	0.100	0.174
Milk lactose (%)	4.64	4.64	0.075	0.993
MUN (%)	13.40	14.45	0.622	0.254
Lactation performance				
4% FCM ^1^	21.61	24.47	0.792	0.024
ECM ^2^	16.23	18.39	0.512	0.006
Milk protein (g/d)	0.79	0.89	0.028	0.022
Milk fat (g/d)	0.82	0.95	0.037	0.031
Milk lactose (g/d)	1.09	1.19	0.050	0.194
Milk protein (g/kg)	33.93	34.85	0.400	0.146
Milk fat (g/kg)	35.06	37.05	0.999	0.174
Milk lactose (g/kg)	46.42	46.43	0.747	0.993
Apparent digestibility				
DM (%)	90.44	87.62	0.881	0.051
EE (%)	82.52	81.42	2.060	0.709
CP (%)	63.55	63.84	2.214	0.928
NDF (%)	73.89	71.25	2.198	0.429
ADF (%)	72.09	71.85	2.427	0.946

^1^ 4% FCM (Fat-Corrected Milk) was calculated as milk yield (kg/d) × [milk fat (%) × 0.15 + 0.4]. ^2^ ECM (Energy-Corrected Milk) was calculated as milk yield (kg/d) × [0.0929 × milk fat (%) + 0.0547 × milk protein (%) + 0.0395 × milk lactose (%)]. Values are presented as mean ± SEM. *p* < 0.05 indicates a significant difference in data between two groups in the same row. Ten samples per group were used for analysis.

**Table 3:** The effect of the partial replacement of alfalfa hay with alfalfa silage on serum biochemical indicators of dairy cows.

Items ^1^	CON	AS	SEM	*p*-Value
TP (g/L)	86.62	88.96	1.600	0.302
ALB (g/L)	38.49	37.08	0.746	0.177
GLB (g/L)	50.38	49.57	1.832	0.766
ALB/GLB	0.79	0.78	0.030	0.778
ALT (U/L)	40.90	38.59	1.624	0.322
AST (U/L)	86.67	91.80	3.171	0.267
AST/ALT	2.59	2.51	0.176	0.757
ALP (U/L)	78.80	73.24	8.045	0.679
BUN (mmol/L)	5.52	5.36	0.242	0.639
GLU (mmol/L)	3.50	3.27	0.182	0.358
TG (mmol/L)	0.16	0.17	0.007	0.191
T-CHO (mmol/L)	4.90	4.87	0.180	0.918

^1^ TP = total protein; ALB = albumin; GLB = globulin; ALT = alanine aminotransferase; AST = aspartate aminotransferase; ALP = alkaline phosphatase; BUN = urea nitrogen; GLU = glucose; TG = triglycerides; T-CHO = total cholesterol. Values are presented as mean ± SEM. *p* < 0.05 indicates a significant difference in data between two groups in the same row. Ten samples per group were used for analysis.

**Table 4:** The effect of the partial replacement of alfalfa hay with alfalfa silage on serum immune parameters of dairy cows.

Items	CON	AS	SEM	*p*-Value
IgG (mg/mL)	6.75	5.84	0.269	0.026
IgA (μg/mL)	2223.63	2018.01	96.547	0.136
IgM (μg/mL)	1425.72	1323.86	62.901	0.259
IFNγ (pg/mL)	727.81	840.99	45.257	0.097
IL1β (pg/mL)	588.70	631.48	24.785	0.235
IL2 (pg/mL)	631.43	735.98	45.290	0.113
IL4 (pg/mL)	43.63	39.16	1.912	0.146
IL6 (pg/mL)	83.41	116.09	7.100	0.003
IL8 (pg/mL)	174.63	183.36	9.589	0.518
TNFα (pg/mL)	147.09	149.93	8.757	0.818

Values are presented as mean ± SEM. *p* < 0.05 indicates a significant difference in data between two groups in the same row. Ten samples per group were used for analysis.

**Table 5:** The effect of the partial replacement of alfalfa hay with alfalfa silage on rumen fermentation parameters of dairy cows.

Items	CON	AS	SEM	*p*-Value
MCP (mg/mL)	1.39	1.24	0.039	0.016
NH_3_-N (mg/dL)	3.64	3.99	0.388	0.516
Acetate (mmol/L)	60.98	63.50	2.450	0.461
Propionate (mmol/L)	23.18	20.30	1.530	0.187
Isobutyrate (mmol/L)	0.58	0.66	0.040	0.193
Butyrate (mmol/L)	9.91	10.96	0.632	0.261
Isovalerate (mmol/L)	1.04	1.03	0.089	0.951
Valerate (mmol/L)	1.42	1.33	0.086	0.471
TVFA (mmol/L)	100.03	96.51	3.167	0.428
Acetate/Propionate	2.78	3.17	0.200	0.190

Values are presented as mean ± SEM. *p* < 0.05 indicates a significant difference in data between two groups in the same row. Ten samples per group were used for analysis.

## Data Availability

The datasets generated and/or analyzed during the current study are available in the NCBI repository, https://dataview.ncbi.nlm.nih.gov/object/PRJNA1277443?reviewer=r3pmcq6cku1cjn94fb23meq3q1 (accessed on 18 June 2025), accession number: PRJNA1277443.

## Abbreviations

The following abbreviations are used in this manuscript:

CP	crude protein
EE	ether extract
NDF	neutral detergent fiber
ADF	acid detergent fiber
DMI	dry matter intake
VFAs	volatile fatty acids
TMR	total mixed ration
TP	total protein
ALB	albumin
GLB	globulin
ALT	alanine aminotransferase
AST	aspartate aminotransferase
ALP	alkaline phosphatase
BUN	urea nitrogen
GLU	glucose
TG	triglycerides
T-CHO	total cholesterol
IgG	immunoglobulin G
IgA	immunoglobulin A
IgM	immunoglobulin M
IFN-γ	interferon-γ
IL1β	interleukin-1β
IL-2	interleukin-2
IL-6	interleukin-6
IL-8	interleukin-8
TNF-α	tumor necrosis factor-alpha
FCM	Fat-Corrected Milk
ECM	Energy-Corrected Milk
MCP	microbial crude protein
NH_3_-N	ammonia nitrogen
